# Baicalin Modulates Inflammatory Response of Macrophages Activated by LPS via Calcium-CHOP Pathway

**DOI:** 10.3390/cells11193076

**Published:** 2022-09-30

**Authors:** Hyo-Jin An, Ji-Young Lee, Wansu Park

**Affiliations:** 1 Department of Pharmacology, College of Korean Medicine, Sangji University, Wonju 26339, Korea; 2 Department of Pathology, College of Korean Medicine, Gachon University, Seong-nam 13120, Korea

**Keywords:** baicalin, anti-inflammation, macrophage, cytokine, chop, lipopolysaccharide, nitric oxide, calcium, p38 MAPK

## Abstract

Studies on natural products that can alleviate the inflammatory response of macrophages caused by endotoxin (lipopolysaccharide) continue. This study investigated the anti-inflammatory activity of baicalin related to macrophage activation caused by lipopolysaccharide (LPS). Baicalin is a flavone glycoside found in plants such as *Scutellaria baicalensis* and *Scutellaria lateriflora* belonging to the genus *Scutellaria*. The multiplex cytokine assay (MCA), Griess reagent assay, fluo-4 calcium assay, dihydrorhodamine 123 (DHR123) assay, quantitative RT-PCR, and flow cytometry were performed using RAW 264.7 mouse macrophages. The MCA revealed that baicalin significantly decreased the production of interleukin (IL)-6, granulocyte colony-stimulating factor (G-CSF), vascular endothelial growth factor (VEGF), macrophage inflammatory protein (MIP)-1α, MIP-1β, MIP-2, and RANTES in LPS-stimulated RAW 264.7 macrophages at concentrations of 10, 25, and 50 μM. The DHR123 assay showed that baicalin significantly inhibited reactive oxygen species generation in LPS-stimulated RAW 264.7 macrophages. Flow cytometry revealed that baicalin significantly reduced the levels of phosphorylated p38 MAPK and Fas in LPS-stimulated RAW 264.7 macrophages. Baicalin also inhibited the mRNA expression levels of inflammatory genes such as *Chop*, *Fas*, *Nos2*, *Ptgs2*, *Stat1*, *c-Jun*, *c-Fos*, and *At1a*. The IC_50_ values of baicalin for IL-6, TNF-α, G-CSF, VEGF, interferon gamma-induced protein 10 (IP-10), leukemia inhibitory factor (LIF), lipopolysaccharide-induced CXC chemokine (LIX), MIP-1α, MIP-1β, MIP-2, RANTES, nitric oxide, intracellular calcium, and hydrogen peroxide were 591.3, 450, 1719, 27.68, 369.4, 256.6, 230.7, 856.9, 1326, 1524, 378.1, 26.76, 345.1, and 32.95 μM, respectively. Baicalin modulated the inflammatory response of macrophages activated by LPS via the calcium-CHOP pathway.

## 1. Introduction

Despite the development of many types of powerful antibiotics, bacterial infections still threaten human health [1,2,3]. Among the disease-causing pathogens, bacteria (eubacteria) and archaebacteria are single-celled organisms [4,5]. Among the various pathogenic bacteria, *Escherichia coli*, *Hemophilus influenza*, and *Pseudomonas aeruginosa* are Gram-negative bacteria (GNB) [6]. GNB have inner and outer membranes with peptidoglycan cell walls between them. Lipopolysaccharide (LPS) makes up the outer membrane of GNB. LPS is composed of an inner core of polysaccharides, lipid A, an outer core of polysaccharides, and O antigens [7,8,9].

When the cell walls of GNB are destroyed or when bacteria die, the endotoxin LPS is released. Unlike exotoxins, known to possess strong immunogenicity, endotoxins exhibit low immunogenicity and are heat-stable. When GNB that invade the human body are phagocytized and destroyed by innate immune cells such as macrophages, the LPS contained within them is released and can bind to specific receptors on macrophages and B cells. When lipid A stimulates immune cells, the production of tumor necrosis factors, interleukins, prostaglandins, and colony-stimulating factors is induced, causing side effects such as fever [10,11].

The activation of macrophages by endotoxins is an important pathophysiological mechanism of septic shock [12]. Despite the development of many treatments, sepsis remains a threat to human health. Septic shock is associated with increased mortality. The low immunogenicity of endotoxins makes it difficult to produce preventive vaccines. Hospital admission rates due to bacterial sepsis and the relatively high mortality rate may be related to the low immunogenicity of endotoxins. The mitigation of so-called endotoxemia may help alleviate septic syndrome, since monocytes and macrophages stimulated by endotoxins can exacerbate septic shock by overproducing inflammatory factors, including nitric oxide (NO) [13]. NO and hydrogen peroxide are free radicals and are associated with cellular pathologies such as endoplasmic reticulum (ER) stress, along with intracellular calcium release [14]. NO is useful for removing pathogens such as bacteria. However, the excessive production of NO by innate immune cells such as macrophages in response to infection can cause the side effect of septic shock, which increases blood permeability and lowers blood pressure [15,16]. Cytokines can also activate the immune system to attract immune cells to infected areas, promote antibody production, remove infected cells, promote lymphocyte proliferation and maturity, and moderately regulate excessive immune activity. However, excessive cytokine production can disturb the blood coagulation system and lead to the development of multiple organ failure through disseminated intravascular coagulation, which plays a role in increasing the mortality rate from sepsis [17]. Therefore, the excessive production of NO and cytokines due to bacterial infections and endotoxemia needs to be controlled. Thus, many studies have reported on the efficacy of natural products that can inhibit the hyper-inflammation of LPS-stimulated macrophages [18].

**Figure 1:**
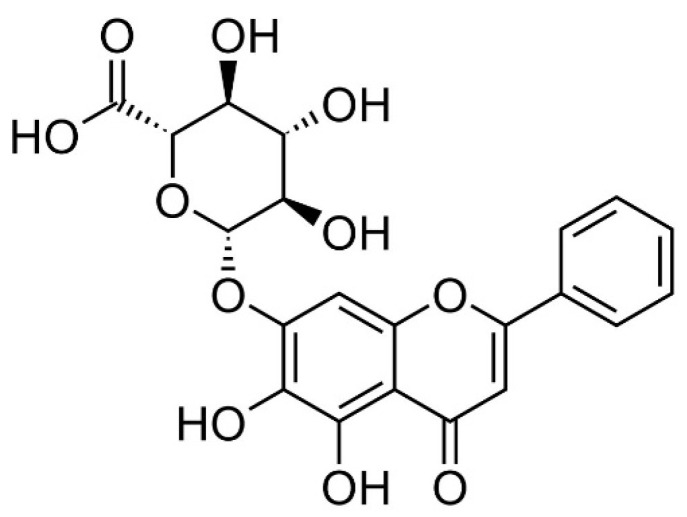
Structural formula of baicalin.

Baicalin (Figure 1) is a flavone glycoside found in plants such as *Scutellaria baicalensis* and *Scutellaria lateriflora* belonging to the genus *Scutellaria* [19]. Baicalin showed anxiolytic effects without myorelaxant effects in mice [20]. Additionally, baicalin has anti-oxidative, anti-inflammatory, and anti-apoptotic effects [21]. Baicalin inhibited myocardial apoptosis and inflammation via Akt/NF-κB signaling, as well as macrophage polarization via the JAK/STAT pathway [21]. In this study, the action of baicalin on the hyper-inflammatory reaction of macrophages stimulated by LPS was investigated. Since inflammatory mediators, especially cytokines, play an important role in inflammatory reactions, this study investigated the production of various cytokines (polypeptide growth factors), chemotactic cytokines, and growth factors, and specifically, cytokines such as interleukin (IL)-6, granulocyte colony-stimulating factor (G-CSF), vascular endothelial growth factor (VEGF), macrophage inflammatory protein (MIP)-1α, MIP-1β, MIP-2, RANTES, interferon gamma-induced protein 10 (IP-10), leukemia inhibitory factor (LIF), lipopolysaccharide-induced CXC chemokine (LIX), IL-10, and tumor necrosis factor (TNF)-α. Additionally, the expression of inflammation-related genes was also investigated. The data showed that baicalin significantly inhibited the production of IL-6, G-CSF, VEGF, MIP-1α, MIP-1β, MIP-2, and RANTES, as well as the transcription of *Chop*, *Fas*, *Nos2*, *Ptgs2*, *Stat1*, *c-Jun*, *c-Fos*, and *At1a* genes in LPS-stimulated RAW 264.7 cells. Additionally, p38 MAPK phosphorylation and Fas levels were significantly decreased in RAW 264.7 cells by baicalein treatment. Such anti-inflammatory effects of baicalin might be through the calcium-CHOP signaling pathway rather than the generally known NF-κB pathway.

## 2. Materials and Methods

The materials and experimental methods used in this study were mostly described in previous studies [22,23]. Detailed explanations are provided in the Supplementary File.

### 2.1. Materials

Dulbecco’s modified Eagle medium (DMEM), baicalin, and other chemicals were obtained from Millipore (Billerica, MA, USA).

### 2.2. Cell Culture and Cell Viability

RAW 264.7 cell lines (second passage) were obtained from the Korea Cell Line Bank (Seoul, Korea). Since mouse macrophages have been widely used for research on the ER stress-related CHOP pathway, RAW 264.7 macrophages were used in this study, not human-derived macrophages. Most human-derived macrophage cell lines in biomedical studies are considered to be differentiated from human-derived monocytes with chemical reactants. Thus, in this study, mouse macrophages were used in order to avoid additional biological changes in the cell lines. It is because biological changes in chemicals-differentiated cells can confuse the interpretation of the action of anti-inflammatory candidate materials. Cell viability was accessed by a modified MTT assay as described previously (Kim et al., 2020). Briefly, the viability of RAW 264.7 cells incubated with 10 and 50 μM baicalin for 24 h were 104.57 ± 0.57% and 114.65 ± 0.11%, respectively, of the normal group treated with media only. Therefore, in subsequent experiments, the cells were treated with a range of baicalin concentrations (10–50 μM).

### 2.3. NO Production

NO production in RAW 264.7 cells (1 × 10^4^ cells/well) was measured after 24 h of treatment with baicalin by the Griess reagent assay (Thermo Fisher Scientific, Waltham, MA, USA) [22,23]).

### 2.4. Intracellular Calcium Release

Intracellular calcium release in RAW 264.7 (1 × 10^5^ cells/well) cells was measured after 24 h of treatment with baicalin using the fluo-4 calcium assay kit (Thermo Fisher Scientific) [22,23].

### 2.5. Hydrogen Peroxide Production

Hydrogen peroxide production in RAW 264.7 cells (1 × 10^4^ cells/well) was measured after 24 h and 48 h of treatment with baicalin by the dihydrorhodamine 123 assay [24].

### 2.6. Multiplex Cytokine Assay

The levels of cytokines released from RAW 264.7 (1 × 10^4^ cells/well) were evaluated after 24 h treatment with baicalin using MILLIPLEX MAP Mouse Cytokine/Chemokine Magnetic Bead Panel kits (Millipore) and a Bio-Plex 200 suspension array system (Bio-Rad, Hercules, CA, USA) [22,23]. The cytokine group in this study included not only cytokines that mainly act on the removal of infectious agents, but also inflammatory mediators that act on leukocyte migration and vascular permeability.

### 2.7. Quantitative RT-PCR

Total RNA was isolated from RAW 264.7 cells (1 × 10^6^ cells/well) after 18 h of treatment with baicalin using a NucleoSpin RNA kit (Macherey-Nagel, Duren, Germany). The quality of the RNA was measured with an Experion Automatic Electrophoresis System (Bio-Rad). Then, cDNA was synthesized using the extracted RNA and an iScript cDNA Synthesis kit (Bio-Rad). The transcription levels of *Chop*, *Fas*, *Nos2*, *Ptgs2*, *Stat1*, *c-Jun*, *c-Fos*, *At1a*, and *β-Actin* (internal control) were evaluated by quantitative RT-PCR using a Bio-Rad CFX 96 (Bio-Rad) [22,23]. The Gene Bank Accession numbers of the primers used in this assay are shown in Table 1.

### 2.8. Flow Cytometric Analysis of Phosphorylated P38 MAPK and Fas

The levels of phosphorylated P38 MAPK and Fas were evaluated via flow cytometry using an Attune NxT flow cytometer (Thermo Fisher Scientific) as described previously [22,23]. Briefly, after 18 h of treatment with baicalin, RAW 264.7 cells (1 × 10^6^ cells/well) were stained with Fixable Viability Dye eFluor 520 (eBioscience 65-0867-18), phospho-P38 MAPK antibody (T180/Y182) (eBioscience 17-9078-42), Fas (APO-1) (eBioscience 12-0951-83), mouse IgG1 kappa isotype control (eBioscience 12-4714-81), and mouse IgG2b kappa isotype control (eBioscience 12-4732-81). After washing the cells with staining buffer, the levels of phosphorylated P38 MAPK and Fas were analyzed with an Attune NxT flow cytometer using Attune NxT software (Thermo Fisher Scientific).

### 2.9. Statistical Analyses

The data are presented as means ± standard deviation of three independent experiments. All data were analyzed by one-way analysis of variance (ANOVA) followed by Tukey’s multiple comparison test using GraphPad Prism (version 4) (GraphPad Software, San Diego, CA, USA). IC_50_ values were also calculated using GraphPad Prism.

## 3. Results

### 3.1. Effect of Baicalin on Cell Viability

The viability of RAW 264.7 macrophages incubated with baicalin at concentrations of 10 and 50 μM for 24 h was 104.57 ± 0.57% and 114.65 ± 0.11%, respectively, of the normal group treated with media only. This means that baicalin was not toxic to cells at a concentration of up to 50 μM. Natural products that are toxic to macrophages are likely to weaken immune function. Thus, if baicalin can regulate the production of inflammatory mediators such as NO and cytokines without causing toxicity to RAW 264.7 cells (i.e., mouse macrophages), it can provide a clue to the development of safer anti-inflammatory drugs.

### 3.2. Effect of Baicalin on NO Production

**Figure 2:**
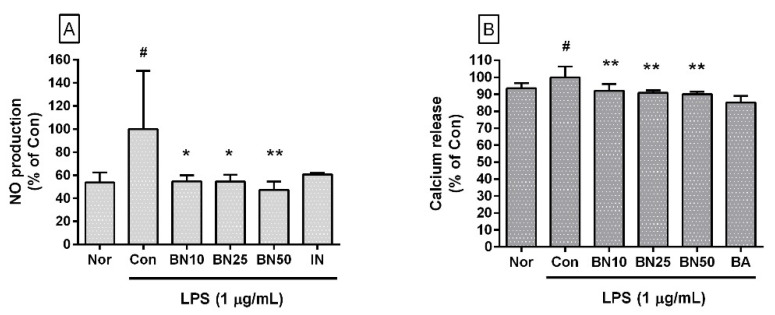
Effect of baicalin on nitric oxide (NO) production (**A**) and calcium release (**B**) in RAW 264.7 cells. Values are the mean ± SD of three independent experiments. Nor, normal group incubated with media only; Con, control group treated with 1 µg/mL of lipopolysaccharide (LPS) only; BN10, BN25, and BN50 indicate 10, 25, and 50 µM of baicalin, respectively; IN, indomethacin (0.5 µM); BA, baicalein (25 µM). # *p* < 0.5 vs. Nor; * *p* < 0.05 vs. Con; ** *p* < 0.01 vs. Con.

Baicalin significantly decreased NO levels in LPS-stimulated RAW 264.7 cells (IC_50_: 26.76 μM) (Figure 2A). The NO levels produced by LPS-stimulated RAW 264.7 macrophages incubated with baicalin at concentrations of 10, 25, and 50 μM for 24 h were 54.71 ± 5.24%, 54.63 ± 5.65%, and 47.42 ± 7.05%, respectively, of the control group treated with LPS only. NO production by macrophages in the immuno-inflammation process of the innate immune system in response to infection has many net functional aspects. However, excessive production can damage surrounding tissues and decrease blood pressure due to increased vascular permeability. Therefore, the inhibitory effect of baicalin on excessive NO production of macrophages in this experiment can be interpreted as alleviating the excessive increase in reactive nitrogen species due to infections such as endotoxemia.

### 3.3. Effect of Baicalin on Intracellular Calcium Release

Baicalin significantly inhibited intracellular calcium release in LPS-stimulated RAW 264.7 cells (IC_50_: 345.1 μM) (Figure 2B). The levels of intracellular calcium release in LPS-stimulated RAW 264.7 cells incubated with baicalin at concentrations of 10, 25, and 50 μM for 24 h were 92.12 ± 3.94%, 90.80 ± 1.66%, and 89.99 ± 1.58%, respectively, of the control group treated with LPS only. Although the exact mechanism involved in the increase in cytoplasmic calcium ion concentrations has not yet been fully identified, increases in cytoplasmic calcium ions are linked to ER stress, the activation of mitogen-activated protein kinase cascades, and the activation of transcription factors such as CHOP (GADD153) and activator protein 1, which might amplify infectious inflammatory responses such as the production of inflammatory cytokines and the expression of Fas, resulting in oxidative stress in the surrounding local tissues and pyroptosis (i.e., a highly inflammatory form of lytic programmed cell death) in infected immune cells [14]. Therefore, this result indicates that baicalin could regulate intracellular calcium signals in LPS-stimulated macrophages.

### 3.4. Effect of Baicalin on Hydrogen Peroxide Production

**Figure 3:**
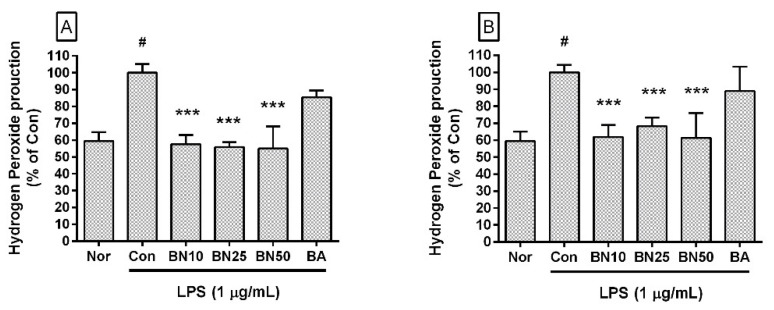
Effect of baicalin treatment of RAW 264.7 cells on hydrogen peroxide production. Macrophages were treated for 24 h (**A**) and 48 h (**B**). Values are presented as mean ± SD of three independent experiments. Nor, normal group incubated with media only; Con, control group treated with 1 µg/mL of LPS only; BN10, BN25, and BN50 are 10, 25, and 50 µM of baicalin, respectively; BA, baicalein (25 µM). # *p* < 0.05 vs. Nor; *** *p* < 0.001 vs. Con.

Baicalin significantly inhibited hydrogen peroxide production in LPS-stimulated RAW 264.7 cells (IC_50_: 32.95 μM for 24 h incubation) (Figure 3). The production levels of hydrogen peroxide in LPS-stimulated RAW 264.7 cells incubated with 10, 25, and 50 μM baicalin for 24 h were 57.59 ± 5.47%, 55.89 ± 2.83%, and 54.99 ± 13.07%, respectively, of LPS treatment alone. After 48 h of incubation, the levels of hydrogen peroxide produced by LPS-stimulated RAW 264.7 cells incubated with 10, 25, and 50 μM baicalin were 61.89 ± 6.98%, 68.28 ± 5.11%, and 61.25%± 14.82%, respectively, of LPS treatment alone. Like reactive nitrogen species, reactive oxygen species play conflicting roles, both removing sources of infection that have invaded human cells and causing oxidative stress in tissues around the infected site. Therefore, these experimental results suggest that baicalin could weaken the ability to remove infectious pathogens in addition to alleviating oxidative stress in macrophages stimulated by LPS.

### 3.5. Effect of Baicalin on Cytokine Production

**Figure 4:**
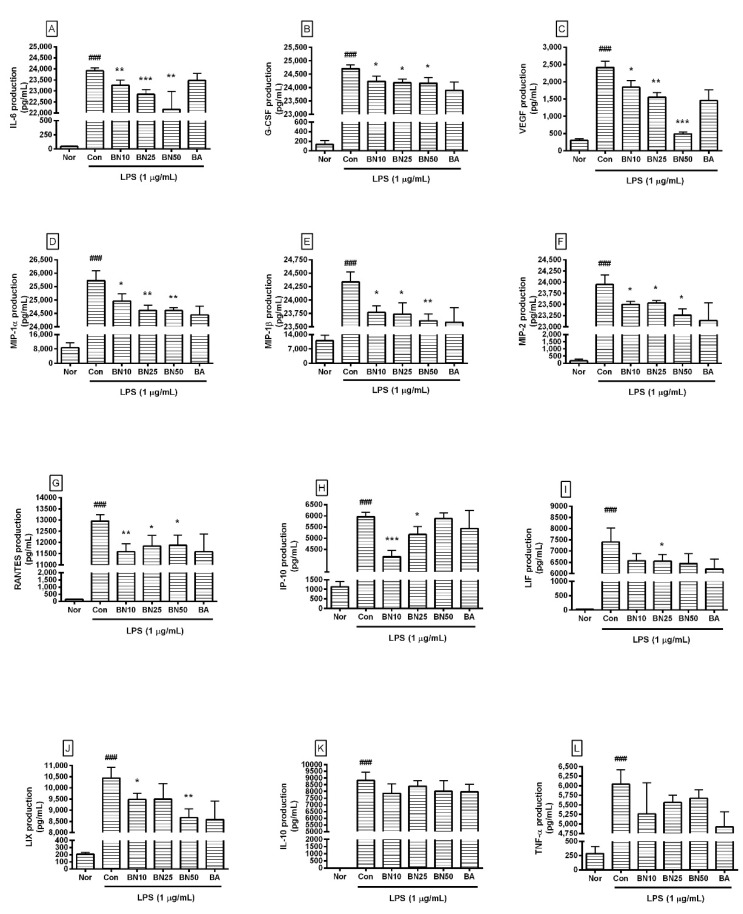
Production of IL-6 (**A**), G-CSF (**B**), VEGF (**C**), MIP-1α (**D**), MIP-1β (**E**), MIP-2 (**F**), RANTES (**G**), IP-10 (**H**), LIF (**I**), LIX (**J**), IL-10 (**K**), and TNF-α (**L**) in RAW 264.7. Values are presented as mean ± SD of three independent experiments. Nor, normal group incubated with media only; Con, control group treated with 1 µg/mL of LPS only; BN10, BN25, and BN50 are 10, 25, and 50 µM of baicalin, respectively; BA, baicalein (25 µM). ### *p* < 0.001 vs. Nor; * *p* < 0.05 vs. Con; ** *p* < 0.01 vs. Con; *** *p* < 0.001 vs. Con.

Baicalin at concentrations of 10, 25, and 50 μM significantly decreased the production of IL-6, G-CSF, VEGF, MIP-1α, MIP-1β, MIP-2, and RANTES in LPS-stimulated RAW 264.7 cells (Figure 4). In detail, the production levels of IL-6 in LPS-stimulated RAW 264.7 cells treated with 10, 25, and 50 µM baicalin were 97.27 ± 0.94%, 95.53 ± 0.89%, and 92.67%± 3.42%, respectively, of the levels in cells treated with LPS alone. The production levels of G-CSF were 97.28 ± 0.65%, 96.97 ± 0.53%, and 96.93 ± 0.81%, respectively, and those of VEGF were 76.27 ± 8.31%, 64.42 ± 5.75%, and 20.34 ± 2.5%, respectively. MIP-1α levels were 97.05 ± 1.03%, 95.69 ± 0.75%, and 95.66 ± 0.43%, respectively; MIP-1β levels were 97.67 ± 0.51%, 97.52 ± 0.88%, and 97.03 ± 0.53%, respectively; and MIP-2 levels were 98.13 ± 0.31%, 98.27 ± 0.24%, and 97.14 ± 0.58%, respectively. RANTES levels were 89.39 ± 2.79%, 91.31 ± 3.66%, and 91.68 ± 3.52%, respectively. LIF levels were 88.8 ± 4.1%, 88.6 ± 3.88%, and 87.16 ± 5.87%, respectively, and LIX levels were 90.87 ± 2.69%, 91.03 ± 6.68%, and 83.05 ± 3.75%, respectively. TNF-α levels were 84.6 ± 13.08%, 89.41 ± 3.05%, and 91.2 ± 3.44%, respectively, and IP-10 levels were 69.93 ± 4.72%, 86.7 ± 5.89%, and 98.72 ± 4.09%, respectively. IL-10 levels were 89.21 ± 7.86%, 95.2 ± 4.87%, and 90.94 ± 9.11%, respectively. However, IP-10 did not show a dose-dependent decrease, and LIF, IL-10, and TNF-α did not show significant changes, which were shortcomings of this study. Baicalin exhibited IC_50_ values of 591.3, 450, 1719, 27.68, 369.4, 256.6, 230.7, 856.9, 1326, 1524, and 378.1 µM for IL-6, TNF-α, G-CSF, VEGF, IP-10, LIF, LIX, MIP-1α, MIP-1β, MIP-2, and RANTES, respectively. These results suggest that baicalin exerted anti-inflammatory effects in LPS-stimulated RAW 264.7 cells by modulating the excessive levels of various cytokines, ameliorating the hyper-inflammatory reaction caused by endotoxemia. However, since the IC_50_ values for G-CSF, MIP-1β, and MIP-2 were much higher than those for other cytokines, it seems that higher concentrations of baicalin are needed to exert significant effects on G-CSF, MIP-1β, and MIP-2. Further research is needed to verify such prominent differences in IC_50_ values.

### 3.6. Effect of Baicalin on mRNA Expression Levels of Inflammatory Genes in RAW 264.7

**Figure 5:**
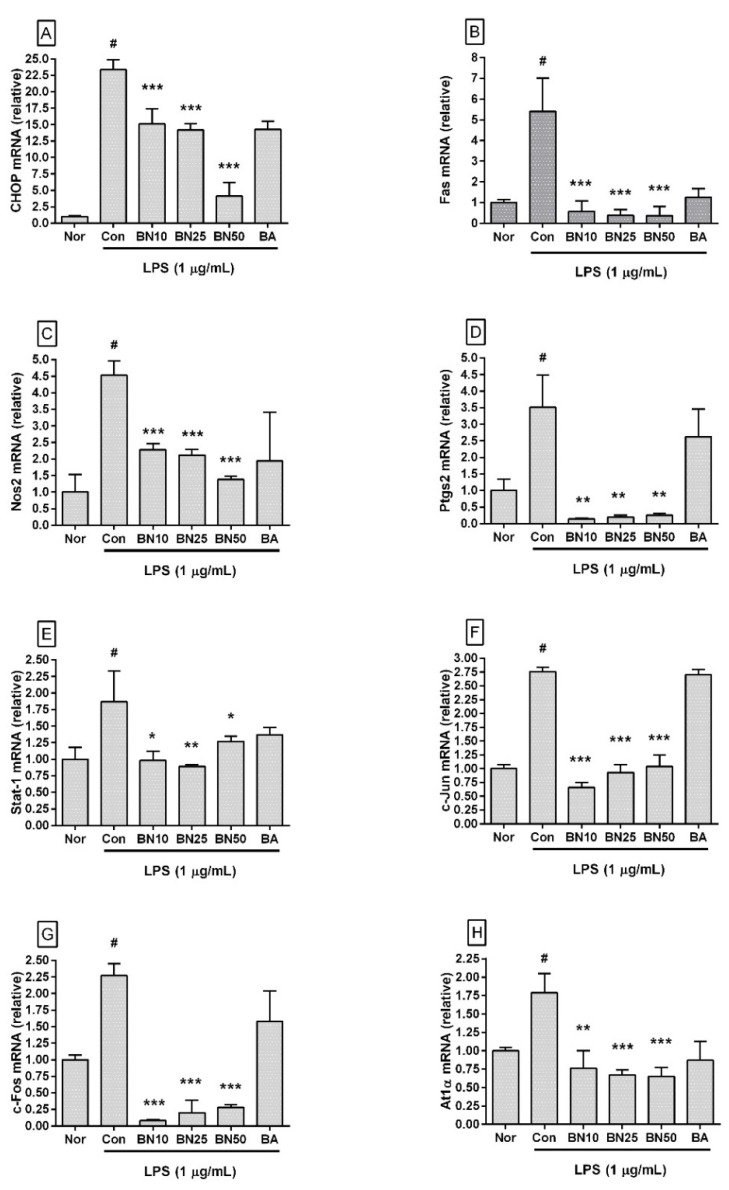
Effect of baicalin on the mRNA expression levels of *Chop* (**A**), *Fas* (**B**), *Nos2* (**C**), *Ptgs2* (**D**), *Stat-1* (**E**), *c-Jun* (**F**), *c-Fos* (**G**), and *At1α* (**H**) in RAW 264.7 macrophages. Values are presented as the mean ± SD of three independent experiments. Nor, normal group incubated with media only; Con, control group treated with 1 µg/mL of LPS only; BN10, BN25, and BN50 are 10, 25, and 50 µM of baicalin, respectively; BA, baicalein (25 µM). # *p* < 0.001 vs. Nor; * *p* < 0.05 vs. Con; ** *p* < 0.01 vs. Con; *** *p* < 0.001 vs. Con.

Baicalin significantly decreased the transcriptional levels of *Chop*, *Fas*, *Nos2*, *Ptgs2*, *Stat1, c-Jun*, *c-Fos*, and *At1a* genes in LPS-stimulated RAW 264.7 (Figure 5). In detail, the *Chop* transcription levels in RAW 264.7 incubated with 10, 25, and 50 µM baicalin were 64.65 ± 10.04%, 60.66 ± 4.3%, and 17.58 ± 8.79%, respectively, of those in the group treated with LPS alone. *Fas* levels were 10.68 ± 9.35%, 7.04 ± 5.19%, and 6.85 ± 8.0%, respectively, and *Nos2* levels were 50.3 ± 3.97%, 46.86 ± 3.81%, and 30.5 ± 2.25%, respectively. *Ptgs2* levels were 3.91 ± 0.51%, 5.33 ± 1.74%, and 7.2 ± 1.59%, respectively, and *Stat1* levels were 52.38 ± 7.44%, 47.3 ± 1.57%, and 67.54 ± 4.11%, respectively. *c-Jun* levels were 23.94 ± 3.09%, 33.82 ± 5.13%, and 37.62 ± 7.73%, respectively, and *c-Fos* levels were 3.73 ± 0.75%, 8.63 ± 8.29%, and 12.3 ± 1.69%, respectively. *At1α* levels were 42.58 ± 13.61%, 37.69 ± 3.75%, and 36.24 ± 6.7%, respectively. These data indicate that baicalin could inhibit inflammatory reactions in LPS-stimulated RAW 264.7 via a CHOP-related pathway that involved intracellular calcium release modulation.

### 3.7. Effect of Baicalin on Levels of Phosphorylated P38 MAPK and Fas in RAW 264.7 Macrophages

**Figure 6:**
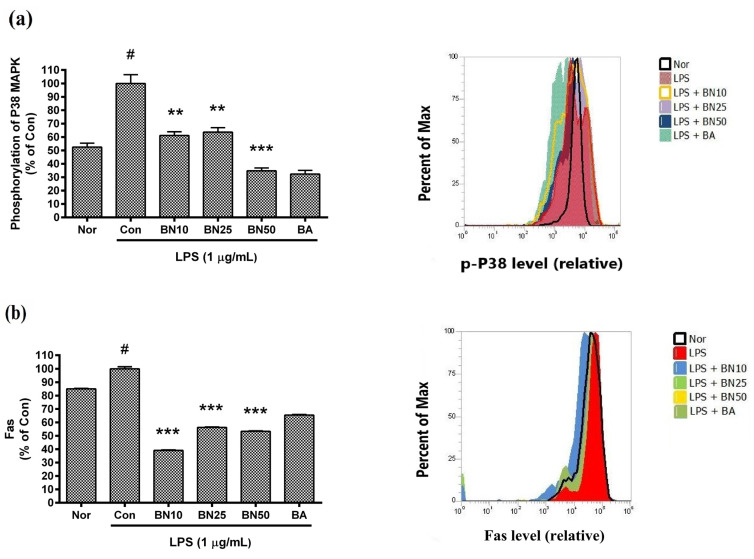
Effect of baicalin on levels of phosphorylated P38 MAPK (**a**) and Fas (**b**) in LPS-stimulated RAW 264.7 macrophages. Levels of phosphorylated P38 MAPK and Fas were measured via flow cytometric analysis. Values are presented as mean ± SD of three independent experiments. Nor, normal group (media only); Con, control group (1 µg/mL of LPS alone). BN10, BN25, and BN50 are 10, 25, and 50 µM of baicalin, respectively; BA, baicalein (25 µM). # *p* < 0.05 vs. Nor; *** *p* < 0.001 vs. Con; ** *p* < 0.01 vs. Con.

Baicalin significantly inhibited the phosphorylation of P38 MAPK and reduced Fas levels in LPS-stimulated RAW 264.7 macrophages (Figure 6). In detail, the P38 MAPK phosphorylation levels in LPS-stimulated RAW 264.7 incubated with 10, 25, and 50 µM baicalin for 18 h were 61.19 ± 2.77%, 63.67 ± 3.27%, and 34.75 ± 2.11%, respectively, of that induced by LPS treatment alone. The Fas levels in LPS-stimulated RAW 264.7 incubated with 10, 25, and 50 µM baicalin for 18 h were 39.06 ± 0.42%, 56.21 ± 0.41%, and 53.37 ± 0.3%, respectively, of that induced by LPS. The results indicate that baicalin modulated macrophage activation via p38 MAPK signaling.

## 4. Discussion

Baicalin, a glucuronide of baicalein, is a key flavonoid found in plants such as *Scutellaria baicalensis* and *Scutellaria lateriflora* and belonging to the genus *Scutellaria*, [20]. Baicalin is known to be a positive allosteric modulator of the benzodiazepine site of the GABA_A_ receptor [20]. It exerted anxiolytic effects without showing myorelaxant effects in mice [21]. Like other flavonoids, baicalin has various pharmacological activities and anti-inflammatory effects. Duan et al. (2021) reported that baicalin could inhibit ferroptosis in nerve growth factor-differentiated pheochromocytoma cells (i.e., PC12 cells) induced by RSL3, hemin, and erastin without apparent toxicity to the liver or kidneys of mice. This anti-ferroptotic effect might be due to its ability to decrease lipid reactive oxygen species in PC12 cells [25]. In the present study, baicalin at concentrations up to 50 μM was found to be toxic to RAW 264.7 macrophages. Some anti-inflammatory drugs are cytotoxic to macrophages, causing concerns that they might weaken immune function, even though they exhibit strong anti-inflammatory effects. Since baicalin could regulate the production of inflammatory mediators without causing toxicity to macrophages, it has a high potential for development as a safer anti-inflammatory drug.

**Figure 7:**
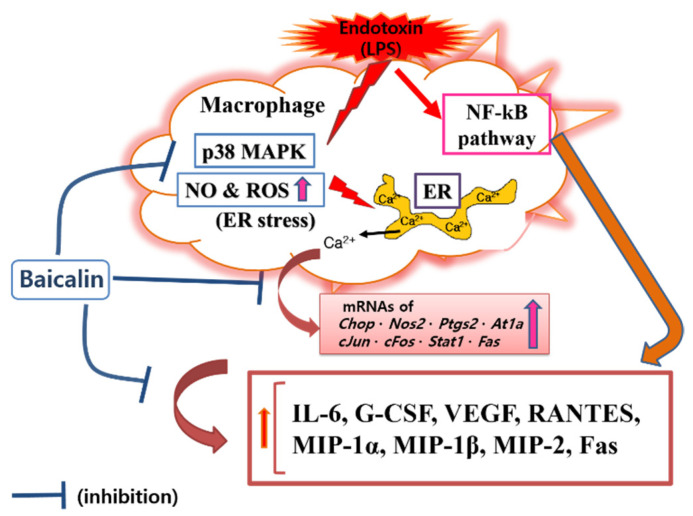
Proposed mechanisms underlying the inhibitory effect of baicalin in LPS-activated RAW 264.7 macrophages via the calcium-CHOP pathway rather than the NF-κB pathway.

In this study, baicalin exhibited IC_50_ values of 26.76, 345.1, and 32.95 μM for NO, intracellular calcium, and hydrogen peroxide, respectively. These results mean that baicalin could relieve oxidative stress in endotoxin-stimulated macrophages by inhibiting the production of reactive nitrogen and reactive oxygen species, along with its ability to modulate calcium signaling. Like reactive nitrogen species, reactive oxygen species play conflicting roles, both removing sources of infection that have invaded human cells and causing oxidative stress in tissues around the infected site. Of course, there is a difference in the role of reactive oxygen species in innate immune cells, such as macrophages, and central nervous brain tissue cells. Thus, the inhibitory effects of baicalin on NO and hydrogen peroxide production in RAW 264.7 cells need to consider its roles in relieving oxidative stress in macrophages due to infection and weakening the ability to remove infectious agents invading cells in endotoxemia. Considering the results of this study showing that baicalin inhibited intracellular calcium production and significantly inhibited the expression of *Chop* genes, it would be reasonable to consider the inhibition of reactive oxygen species and reactive nitrogen species as a mechanism to relieve ER stress in LPS-stimulated macrophages. In fact, ER stress begins with the accumulation of misfolded proteins. It can be worsened by the inadequacy of redox homeostasis caused by changes in the cell environment or stimulation by infectious pathogens [14]. That is, the production of reactive oxygen species due to various stimuli inside and outside the cell can cause ER stress and unfolded protein reactions. In addition, changes in redox homeostasis in the ER can cause ER stress. Conversely, ER stress can increase the production of reactive oxygen species in the ER and mitochondria, with increases in CHOP expression [26,27,28]. In this study, it was not possible to determine whether reactive oxygen species were produced in the ER or mitochondria. However, reactive oxygen species levels changed in response to changes in NO production and calcium concentrations in the cytoplasm. Therefore, the inhibitory effect of baicalin on the production of inflammatory factors could be related to ER stress. Furthermore, it has been already reported that the CHOP-caspase-11 pathway, with the activation of p38 MAPK and STAT-1, plays a key role in the ER stress-related inflammation caused by LPS, which can finally induce Fas and cytokine production [29,30,31,32]. Interestingly, activating protein-1 (AP-1) is necessary for inducing CHOP in ER stress [33]. In this study, since baicalin reduced p38 MAPK phosphorylation, Fas levels, and the expression of *c-Jun* and *c-Fos*, the anti-inflammatory action of baicalin could be said to be achieved via the calcium-CHOP pathway (Figure 7).

Recently, Yan et al. reported that baicalin could decrease cytokine and iNOS mRNA levels in LPS-activated RAW 264.7 cells via the NF-κB pathway [34]. Xu et al. reported that baicalin could decrease iNOS and IL-6 and alleviate post-ischemia/reperfusion myocardial injury [21]. It is well known that LPS triggers macrophage inflammatory reactions through NF-κB signaling [35]. Therefore, many studies showed that various natural products, including baicalin, regulated the inflammatory response of endotoxin-induced macrophages through NF-κB signaling.

In this study, attention was paid to modulating endotoxin-induced inflammatory responses through calcium-CHOP signaling. Since macrophages are most frequently used in in vitro inflammation model studies, the RAW 264.7 macrophage cell line was used in these experiments. CHOP is well known to be an important transcription factor in defective efferocytosis-coupled macrophage apoptosis related to ER stress and unfolded protein responses in advanced atherosclerotic lesions [31]. CHOP-amplified calcium release from ER stores is an important part of ER stress-induced macrophage apoptosis. Our data showed that baicalin decreased the level of cytosolic calcium in LPS-stimulated RAW 264.7 macrophages, which suggests that baicalin plays a role in inhibiting the progression of ER stress. However, the study could not confirm how baicalin affects the activation of calcium/calmodulin-dependent protein kinase II alpha (a key trigger of apoptosis) and cytochrome c release from mitochondria, which might cause the inflammatory cascade of ER stress in activated macrophages. Cardoso et al. reported that AT1R was associated with ER stress and p38 MAPK activation in angiotensin II-induced podocyte apoptosis [36]. The current study showed that baicalin significantly decreased IL-6, G-CSF, VEGF, MIP-1α, MIP-1β, MIP-2, and RANTES levels in LPS-stimulated RAW 264.7 macrophages. Baicalin also significantly decreased the transcription of *Chop*, *Fas*, *Nos2*, *Ptgs2*, *Stat1*, *c-Jun*, *c-Fos*, and *At1a* genes, P38 MAPK phosphorylation, and Fas levels. To reveal the sequential signaling cascade, the expression of inflammatory genes and P38 MAPK phosphorylation were investigated after 18 h of treatment, and cytokines were investigated after 24 h of treatment. The data indicated that baicalin exerted sequential changes in the inflammatory responses of LPS-stimulated RAW 264.7 macrophages. In detail, baicalin exhibited IC_50_ values of 591.3, 450, 1719, 27.68, 369.4, 256.6, 230.7, 856.9, 1326, 1524, and 378.1 µM for IL-6, TNF-α, G-CSF, VEGF, IP-10, LIF, LIX, MIP-1α, MIP-1β, MIP-2, and RANTES, respectively. The shortcoming of this experiment is that the calculated IC_50_ values are very different and high in some cases, but it means that the effect of baicalin on the production of cytokines is not uniform and individual. IP-10 did not show a dose-dependent decrease, and LIF, IL-10, and TNF-α did not show significant changes, which are also shortcomings of this study. When referring to prior studies, the current data indicate that baicalin can exert anti-inflammatory effects in LPS-activated RAW 264.7 cells via the calcium-CHOP pathway rather than the generally known NF-κB pathway. It is important to investigate the meaning of the inhibitory effect of baicalin on the production of cytokines in LPS-stimulated macrophages in the course of an infection. Additionally, since disseminated intravascular coagulation is related to the occurrence of free radicals (i.e., NO and hydrogen peroxide) and intracellular calcium release in endotoxemia [37], more detailed research is needed to determine the effects of baicalin on disseminated intravascular coagulation and multiple organ failure associated with free radical production and intracellular calcium release in immune cells.

## 5. Conclusions

Baicalin could significantly modulate increases in levels of NO, Ca^2+^, hydrogen peroxide, IL-6, G-CSF, VEGF, MIP-1α, MIP-1β, MIP-2, and RANTES in LPS-stimulated RAW 264.7 cells via the calcium-CHOP pathway. More detailed research is needed to achieve the clinical use of baicalin.

## Figures and Tables

**Table 1:** Primers used in quantitative RT-PCR.

Gene Name	Gene Bank Accession number
*Chop*	NM_007837
*Fas*	NM_007987
*Nos2*	NM_010927.3
*Ptgs2*	NM_011198
*Stat1*	NM_009283.4
*c-Jun*	NM_010591
*c-Fos*	NM_010234
*At1a*	NM_177322
*β-Actin*	NM_007393.3

## Supplementary Materials

The following supporting information can be downloaded at: https://www.mdpi.com/article/10.3390/cells11193076/s1, Supplementary Material for Materials and Methods.
