# Supplementary material for: Baicalin Modulates Inflammatory Response of Macrophages Activated by LPS via Calcium-CHOP Pathway
Source: Cells. 2022 Sep 30;11(19):3076. doi: 10.3390/cells11193076 (PMC9563021; doi:10.3390/cells11193076)
Supplement: Supplementary file 1 [file cells-11-03076-s001.zip › cells-1891926-supplementary.pdf]

cells

MDPI

Article

Baicalin Modulates Inflammatory Response of Macrophages Activated by LPS via Calcium-CHOP Pathway

Hyo-Jin An [1,+] , Ji-Young Lee [2,+], and Wansu Park [2,*]

[1] Department of Pharmacology, College of Korean Medicine, Sangji University, Wonju, Gangwon-do 26339, Republic of Korea ; hjan@sj.ac.kr
[2] Department of Pathology, College of Korean Medicine, Gachon University, Seong-nam, 13120, Republic of Korea; oxygen1119@naver.com (J.-Y.L.); pws98@gachon.ac.kr (W.P.)
* Correspondence: pws98@gachon.ac.kr; Tel.: 82-31-750-8821
+ H.-J.A. and J.-Y.L. contributed equally to this work.

< Supplementary Material for Materials and Methods>

1. Materials and Methods

The detailed description of the materials and experimental methods used in this study is as follows.

1.1. Materials

Dulbecco's modified Eagle's medium (DMEM), FBS, penicillin, streptomycin, Phosphate-buffered saline(1x), lipopolysaccharide (LPS), baicalin, and other cell culture reagents were purchased from Millipore (Billerica, MA, USA). Griess reagent assay kits and Fluo-4 calcium assay kits were purchased by Thermo Fisher Scientific (Waltham, MA, USA). Dihydrorhodamine 123 assay kits were purchased from Millipore. Multiplex cytokine assay kits were purchased from Millipore. Real-time RT-PCR kits were ordered from Bio-Rad (Hercules, CA, USA). Fixable Viability Dye eFluor 520 (eBioscience 65-0867-18), phospho-P38 MAPK Antibody (T180/Y182) (eBioscience 17-9078-42, Life Technologies Corporation, Carlsbad, CA, USA), Fas (CD95 Anti-body; APO-1) (eBioscience 12-0951-83), Mouse IgG1 kappa Isotype Control (eBioscience 12-4714-81), and Mouse IgG2b kappa Isotype Control (eBioscience 12-4732-81) were obtained from Life Technologies Corporation (Carlsbad, CA, USA). All other solutions for flow cytometric analysis were purchased from Thermo Fisher Scientific.

1.2. Cell culture and cell viability

RAW 264.7 mouse macrophages were obtained from Korea Cell Line Bank (Seoul, Korea). RAW 264.7 cells were cultured with DMEM supplemented with 10% FBS containing 100 U/mL of penicillin and 100 μg/mL of streptomycin at 37 °C in a 5% CO_2 humidified incubator. Before experimental assays, RAW 264.7 were washed with phosphate buffer saline. Briefly, RAW 264.7 were incubated with baicalin at concentrations of 10 and 50 μM for 24 h in 96-well plates ($1x10^4$ cells/well) in order to verify the toxicity of baicalin. After 24 h culture with baicalin, cell viability was confirmed with the tetrazolium-based colorimetric assay (a modified MTT assay). OD was determined at 540 nm with a microplate reader (Bio-Rad, Hercules, CA, USA).

1.3. Quantification of NO Production in RAW 264.7 mouse macrophages

NO concentration in culture medium was determined by the Griess reagent assay. Specifically, after incubating RAW 264.7 ($1x10^4$ cells/well) with LPS and/or baicalin for 24 h, 100 μL of supernatant from each well was mixed with 100 μL Griess reagent in a 96-well plate. After a 15 min incubation at room temperature, optical density was determined at 540 nm with a microplate reader (Bio-Rad, Hercules, CA, USA).

1.4. Ca^{2+} release in RAW 264.7 mouse macrophages

Ca^{2+} release from RAW 264.7 mouse macrophages was measured by Fluo-4 calcium assay kit (Thermo Fisher Scientific). RAW 264.7 mouse macrophages in 96-well plates ($1x10^5$ cells/well) were incubated with LPS and/or baicalin for 18 h at 37 °C. Thereafter, the medium was removed, and the cells were incubated with 100 μL of the Fluo-4 dye loading solution for 30 min at 37 °C. After the incubation, fluorescence intensity in each well was determined spectrofluorometrically (Dynex, West Sussex, UK) with excitation and emission filters of 485 nm and 535 nm, respectively.

1.5. Hydrogen peroxide production in RAW 264.7 mouse macrophages

Hydrogen peroxide production in RAW 264.7 cells ($1x10^4$ cells/well) after 24 h and 48 h treatment was measured by dihydrorhodamine 123 (DHR) assay. During the cellular production of reactive oxygen species, the nonfluorescent DHR was oxidized by H_2O_2 and irreversibly converted to the green fluorescent compound rhodamine 123 (R123). R123 was membrane-impermeable and accumulated in the cells. An aliquot of DHR (to produce a concentration of 10 μM in each well) was added to each 96-well plate and pre-incubated for 30 min at 37 °C. Thereafter, the medium was removed and RAW 264.7 cells were incubated with LPS and/or baicalin for 24 h at 37 °C. After incubation, fluorescence intensities of each well were analyzed by spectrofluorometer (Dynex) with excitation filter 485 nm and emission filter 535 nm.

1.6. Multiplex Cytokine Assay for cytokines production in RAW 264.7 mouse macrophages

Cytokines from RAW 264.7 cells after 24 h treatment were evaluated with MILLIPLEX MAP Mouse Cytokine/Chemokine Magnetic Bead Panel kits (Millipore) and a Bio-Plex 200 suspension array system (Bio-Rad, Hercules, CA, USA). Briefly, RAW 264.7 cells were seeded in 96-well plates ($1x10^4$ cells/well) and treated with LPS and/or baicalin [15]. After 24 h treatment, levels of the following cytokines in each well were analyzed: interleukin (IL)-6; IL-10; tumor necrosis factor (TNF)-α; leukemia inhibitory factor (LIF); lipopolysaccharide-induced CXC chemokine (LIX; CXCL5); granulocyte colony-stimulating factor (G-CSF); macrophage inflammatory proteins (MIP)-1α, MIP-1β, MIP-2; RANTES (CCL5; regulated on activation, normal T cell expressed and secreted); and interferon gamma-induced protein 10 (IP-10; CXCL10). Cytokines were measured using a Luminex assay based on xMAP technology with MILLIPLEX MAP Mouse Cytokine/Chemokine Magnetic Bead Panel kits and a Bio-Plex 200 suspension array system. The assay used in this experiment was designed for the multiplexed quantitative measurement of multiple cytokines in a single well, using as little as 25 μL of cell culture supernatant. Standard curves for each cytokine were generated using the kit-supplied reference cytokine samples. Briefly, the following procedure was performed: after pre-wetting the 96-well plate with Wash Buffer, Wash Buffer in each well was removed using a Handheld Magnetic Separation Block (HMSB). Next, cell culture supernatants from each well were incubated with antibody-conjugated beads on a plate shaker for 2 h at room temperature. After incubation, well contents were gently removed with a HMSB, and the 96-well plate was washed 2 times. Then, 25 μL of Detection Antibodies were added to each well and incubated with agitation on a plate shaker for 1 h at room temperature. Subsequently, 25 μL Streptavidin–Phycoerythrin was added to each well containing the Detection Antibodies and incubated for 30 min with agitation on a plate shaker at room temperature. After incubation, the well contents were gently removed and washed 2 times using a HMSB. Then, 150 μL of Sheath Fluid was added to all wells, and the beads bound to each cytokine were analyzed with a Bio-Plex 200 instrument (Bio-Rad). Raw data (fluorescence intensity) were analyzed using Bio-Plex Manager software (Bio-Rad). Baicalein (25 μM), a well-known anti-inflammatory flavonoid, was used as a positive control.

1.7. Quantitative real time RT-PCR for mRNA Expression in RAW 264.7 mouse macrophages

Total RNA of RAW 264.7 ($1x10^6$ cells/well) was isolated using NucleoSpin RNA kit (Macherey-Nagel, Duren, Germany) after 18 h treatment. Then, cDNA of the RNA samples was synthesized using iScript cDNA Synthesis kit (Bio-Rad) and checked with an Experion Automatic Electrophoresis System (Bio-Rad). The transcription *Chop*, *Fas*, *Nos2*, *Ptgs2*, *Stat1*, *c-Jun*, *c-Fos*, *At1a*, and *β-Actin* (internal control) was evaluated with Quantitative RT-PCR using Bio-Rad CFX 96 (Bio-Rad). The details of the step-by-step experiment are as follows.

1.7.1. Isolation of RNA

RAW 264.7 cells were incubated with LPS and/or baicalin for 18 h in 6-well plates ($1x10^6$ cells/well). Baicalein (25 μM), a well-known anti-inflammatory flavonoid, was used as a positive control. After 18 h incubation, total RNA of each well was isolated using NucleoSpin RNA kit (Macherey-Nagel, Duren, Germany). Briefly, 350 μL Lysis Buffer RA1 and 3.5 μL β-mercaptoethanol was added to the cell pellet and vortexed vigorously to lyse cells. Lysate was cleared by filtration using a NucleoSpin® Filter, then 350 μL ethanol (70%) was added, and mixed by vortexing. The lysate was loaded into the NucleoSpin® RNA Column, and 350 μL Membrane Desalting Buffer was added and centrifuged. 95 μL DNase reaction mixture was applied directly to the center of the silica membrane of the column, followed by incubation at room temperature for 15 min. Samples were washed with Wash Buffer RAW2 and Wash Buffer RA3, and silica membrane was dried. RNA was eluted in 60 μL RNase-free water and centrifuged.

1.7.2. Determination of RNA concentration

RNA concentration was measured using Experion RNA StdSens Analysis kit (Bio-Rad) with the Experion Automatic Electrophoresis System (Bio-Rad). First, the electrodes were cleaned using a cleaning chip filled with 900 µL DEPC-treated water. Then, the Gel-Stain solution was prepared, and 9 µL was added into labeled wells, and the chip was primed. Samples and RNA ladder were loaded into the chip, which was vortexed using the Experion vortex station for 1 min. Then the chip was loaded into the electrophoresis platform and the RNA StdSens Analysis program was run.

1.7.3. cDNA synthesis

cDNA of the RNA samples was produced using iScript cDNA Synthesis kit (Bio-Rad). Briefly, 20 µL complete reaction mixes were prepared with 5x iScript Reaction Mix (4 µL), iScript Reverse Transcriptase (1 µL), Nuclease-free water (variable), and RNA template (variable, 1 µg total RNA). The reaction mix (20 µL) was incubated in a thermal cycler (C1000 Thermal Cycler, Bio-Rad) according to the manufacturer's protocol (priming at 25°C for 5 min, reverse transcription at 46°C for 20 min, and RT inactivation at 95°C for 1 min).

1.7.4. quantitative RT-PCR analysis

Gene expression was measured using quantitative polymerase chain reaction with iQ SYBR Green Supermix (Bio-Rad) using the CFX96 Real-Time PCR Detection System (Bio-Rad). Briefly, a master mix was prepared for all reactions by adding iQ SYBR Green Supermix and Forward/Reverse primers for each target gene. This master mix was thoroughly mixed to ensure homogeneity, and 7 µL was dispensed into the wells of a qPCR plate. 3 µL of cDNA was added to each well; any air bubbles in the vessel bottom were removed, and the PCR plate was loaded into the real-time PCR instrument. PCR was performed using the following protocol: denaturation of DNA at 95°C for 3 min, followed by 40 cycles of 95°C for 10 sec and 55°C for 30 sec. The $2^{-\Delta\Delta Ct}$ cycle threshold method was used to normalize the relative mRNA expression levels to the internal control, *β-actin*. The primers used in this assay are listed in Table 1.

Table 1. Primers used in quantitative real time PCR

Name[1]	Forward Primer (5′–3′)	Reverse Primer (5′–3′)
Chop	CCACCACACCTGAAAGCAG	TCCTCATACCAGGCTTCCA
Fas	CGCTGTTTTCCCTTGCTG	CCTTGAGTATGAACTCTTAACTGTGAG
Nos2	TGGAGGTTCTGGATGAGAGC	AATGTCCAGGAAGTAGGTGAGG
Ptgs2	TCAAACAGTTTCTCTACAACAACTCC	ACATTTCTTCCCCCAGCAA
Stat1	TGAGATGTCCCGGATAGTGG	CGCCAGAGAGAAATTCGTGT
c-Jun	ACTGGGTTGCGACCTGAC	CAATAGGCCGCTGCTCTC
c-Fos	AGAGCGGGAATGGTGAAGA	TCTTCCTCTTCAGGAGATAGCTG
At1a	TCACCTGCATCATCATCTGG	AGCTGGTAAGAATGATTAGG
β-Actin	CTAAGGCCAACCGTGAAAAG	ACCAGAGGCATACAGGGACA

[1]Primer names: C/EBP homologous protein (*Chop*), First apoptosis signal receptor (*Fas*), Nitric oxide synthase 2 (*Nos2*), Prostaglandin-endoperoxide synthase 2 (*Ptgs2*), Signal Transducer and Activator of Transcription 1 (*Stat1*), *c-Jun*, *c-Fos*, Angiotensin II receptor type 1a (*At1a*), and *β-Actin*.

1.8. Flow Cytometric Analysis for the level of Phosphorylated P38 MAPK and Fas

The level of phosphorylated P38 and Fas receptor in LPS-stimulated RAW 264.7 were evaluated via flow cytometry using an Attune NxT flow cytometer (Thermo Fisher Scientific). Briefly, after 18 h of treatment, RAW 264.7 (3 x 10^5 cells/well) were stained with Fixable Viability Dye eFluor 520 (eBioscience 65-0867-18), phospho-P38 Antibody (T180/Y182) (eBioscience 17-9078-42), Fas (CD95 Anti-body; APO-1) (eBioscience 12-0951-83), IgG1 kappa Isotype Control (eBioscience 12-4714-81), and Mouse IgG2b kappa Isotype Control (eBioscience 12-4732-81) according to manufacturer's protocol. Fixable Viability Dye eFluor 520 was used to irreversibly label dead cells prior to cryopreservation, fixation and/or permeabilization procedures. Cells were fixed with Fix Buffer I (Thermo Fisher Scientific), permeabilized with Perm Buffer III (Thermo Fisher Scientific), and stained with fluorescent-labeled antibodies. Stained cells were analyzed on the Attune NxT flow cytometer (Thermo Fisher Scientific). A serial gating strategy used forward scatter versus side scatter plots, forward scatter versus viability stain plots, and the target antibody expression plots. Unstained cells were used as the negative controls for gating. Data were obtained from the mean fluorescent intensities of samples. Details for startup, proper calibration and operation of the Attune can be found in the Attune User Guide (https://assets.thermofisher.com/TFS-Assets/LSG/manuals/100024235_AttuneNxT_HW_UG.pdf). Mouse IgG1 kappa Isotype

Control was used to confirm the specificity of Fas Antibody. Mouse IgG2b kappa Isotype Control was used to confirm the specificity of phospho-p38 MAPK Antibody. For analysis of raw data, Attune NxT software (Thermo Fisher Scientific) was used. Baicalein (25 μM), a well-known anti-inflammatory flavonoid, was used as a positive control.

1.9. Statistical analyses

Data are presented as means ± standard deviation of three independent experiments. All data were analyzed by one-way analysis of variance (ANOVA) test followed by Tukey's multiple comparison test using GraphPad Prism (version 4; GraphPad Software, San Diego, CA, USA).
